# A 13-year journey of a gastric band – ultimate destination terminal jejunum: a case report

**DOI:** 10.1186/s13256-018-1850-5

**Published:** 2018-10-17

**Authors:** Jeannette D Widmer, Stephanie Schade, Markus K Muller

**Affiliations:** Department of Surgery, Kantonsspital Frauenfeld, 8500 Frauenfeld, Switzerland

**Keywords:** Gastric banding, Complication, Erosion, Migration

## Abstract

**Background:**

Laparoscopic adjustable gastric banding has been the gold standard for surgical management of obesity over the last decades in USA and Europe. However, significant complications have been documented due to foreign body placement, including band erosions. Our treatment approach for erosions is rather observant with regular follow-up until the band has sufficiently perforated the gastric wall which facilitates endoscopic removal. Consequences of a not followed-up band erosion may present even after a long time following initial diagnosis with more severe complications.

**Case presentation:**

A 51-year-old Caucasian woman presented to our out-patients’ clinic with a 2-week history of worsening abdominal pain in her left upper quadrant, exacerbated by abdominal flexion and extension maneuvers.

Here we describe a case involving gastric penetration and subsequent downward migration of a band into distal jejunum causing small bowel obstruction, which occurred more than 10 years following initial diagnosis of erosion. The perforation was missed due to cessation of endoscopic follow-up.

**Conclusion:**

Prospective and long-term follow-up is mandatory in those with partial band erosion to avoid further complications.

## Background

At the end of the 1970s, the idea of a restrictive device to limit nutritional intake arose, with the intent of assisting in weight loss for obese patients. To this effect, Wilkinson developed a non-adjustable band that could be placed around the upper part of the stomach by open surgery [1]. Silicone gastric bands showed the best results with far fewer adhesions and tissue reactions than other materials under trials. Further advances included “adjustability” to the band device via a subcutaneous port system, in addition to a laparoscopic approach to implantation [2]; these advances collectively led to the procedure becoming a gold standard for surgical management of obesity for years.

Although preliminary studies presented promising short-term results, longer-term data are more modest, illustrating a wide variation in weight loss, with a proportion of patients being classified as treatment failures [3]. Moreover, significant complications have been documented due to foreign body placement; including pouch dilatation, band erosions, intolerance, leakage, and slippage. Here we report a case involving gastric penetration and subsequent downward migration of a band into distal jejunum, causing small bowel obstruction over a decade following initial diagnosis of erosion.

## Case presentation

A 51-year-old Caucasian woman presented to our out-patients’ clinic with a 2-week history of worsening abdominal pain in her left upper quadrant, exacerbated by abdominal flexion and extension maneuvers. She described symptoms as intermittent, and accompanied by loss of appetite, nausea, and having a “feculent” taste in her mouth. Her previous medical history was notable for an elevated body mass index (BMI) of 41 kg/m^2^, and in the year 2000 for laparoscopic adjustable gastric band (LAGB) implantation. She experienced weight loss of 30 kg after her original procedure, although 3 years later a partial perforation of the band into her stomach developed (confirmed endoscopically). Over the subsequent years, she required serial endoscopic follow-ups until 2005 when the band was covered by the gastric mucosa only on one third of its surface. From then on and for unknown reasons the endoscopic follow-up ceased. She remained asymptomatic and regained weight up to a BMI of 36.3 kg/m^2^ until her current presentation.

**Fig. 1:**
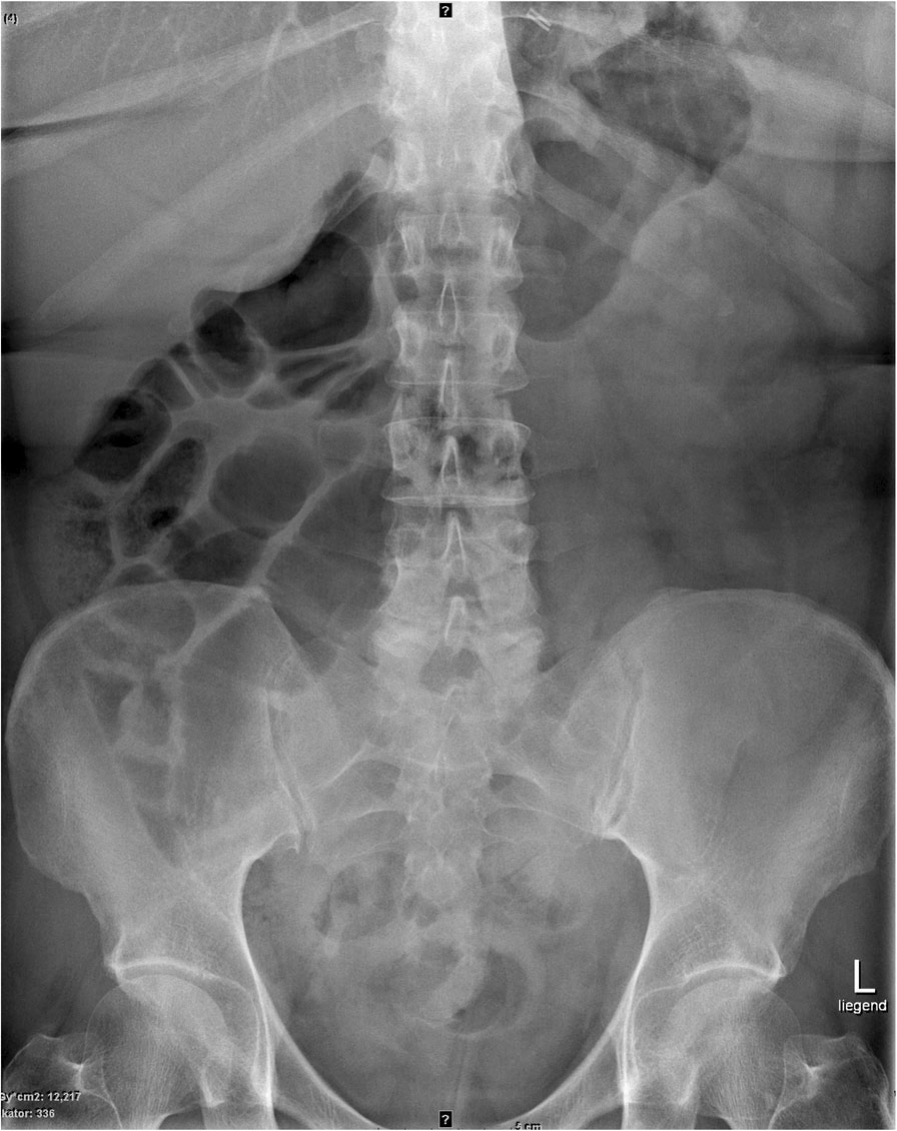
Plain X-ray showing dilated small bowel loops

**Fig. 2:**
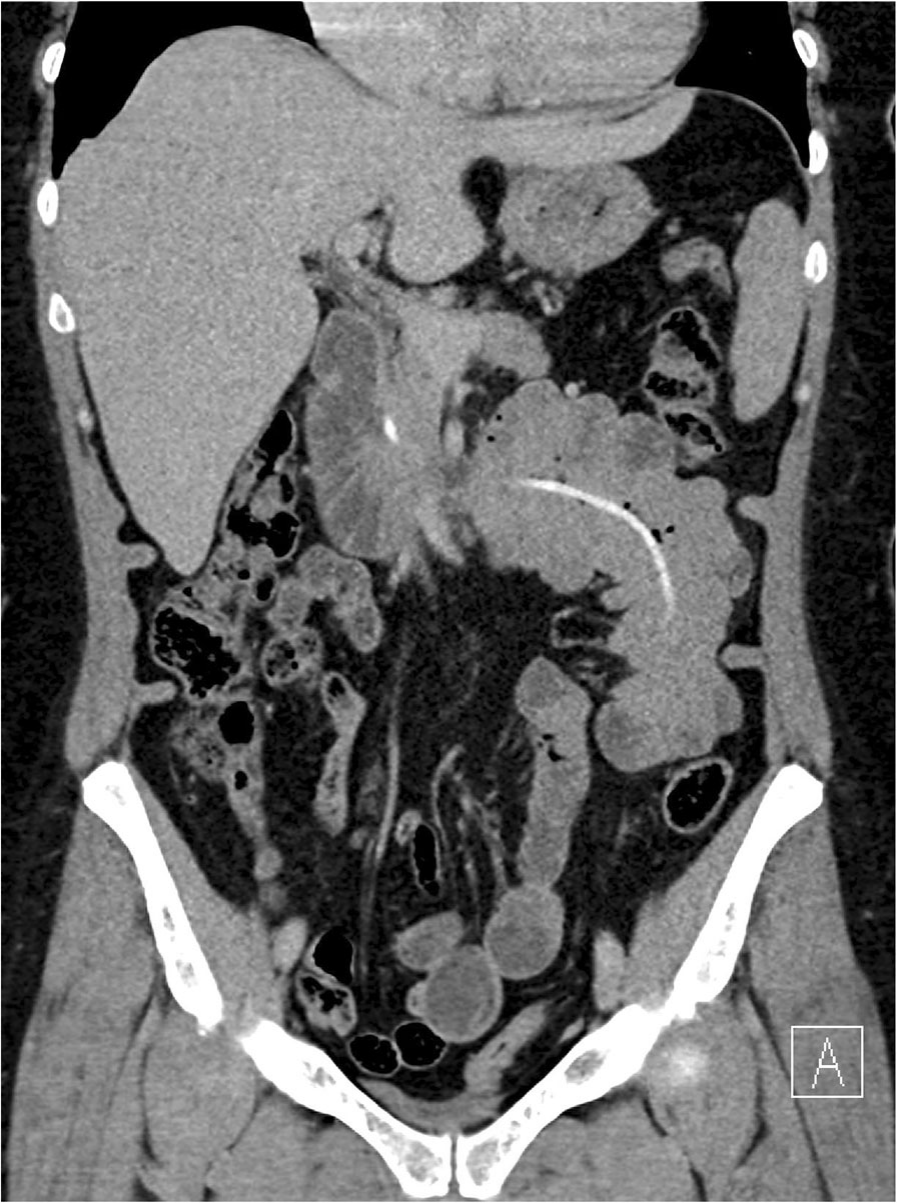
Computed tomography scan with jejunum threaded on the catheter

A clinical examination revealed left-sided abdominal tenderness without signs of abdominal guarding. The blood results were unremarkable, except white blood cells (WBC) with 11.3 G/l. A plain abdominal radiograph showed signs of small bowel obstruction (Fig. 1); and abdominal computed tomography scanning revealed intraluminal migration of the gastric band into the distal jejunum – still connected to the subcutaneous reservoir – folding the intestine on the catheter like a hand organ instrument (Fig. 2).

**Fig. 3:**
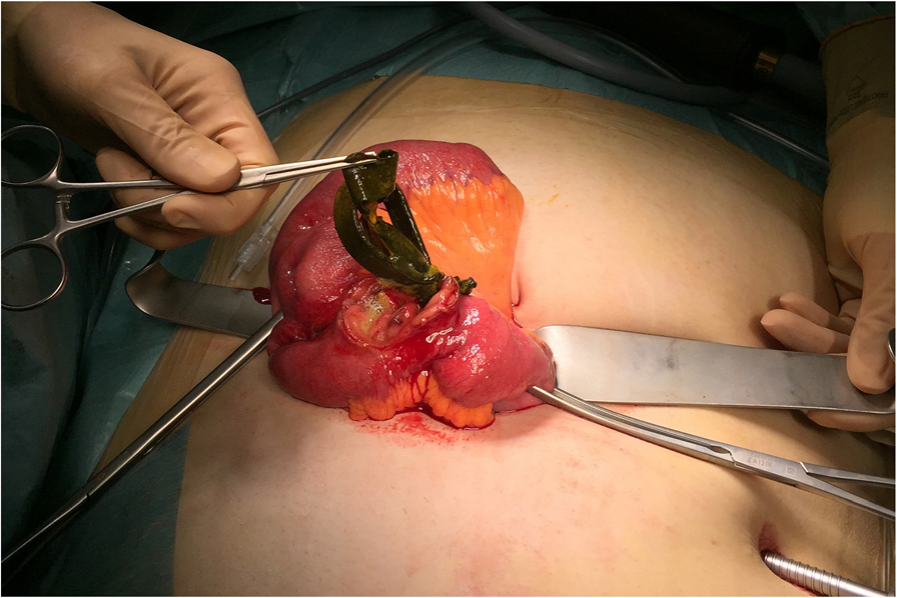
Extraction of the band out of the jejunum

After an unsuccessful attempt to remove the band endoscopically, our patient underwent a diagnostic laparoscopy, which showed that the gastric band was impacted in the distal jejunum causing obstruction. An enterotomy was performed via umbilical mini-laparotomy, and the partially digested silicon band was retrieved (Fig. 3). The involved jejunal segment was resected due to its conglomerate formation with possible stenosis, followed by an end-to-end anastomosis. The operation was completed by removal of the port with the remaining catheter through the original port-site incision. The catheter entrance into the stomach was left untouched, and our patient’s recovery was uneventful. She explicitly did not want a conversion about an alternative bariatric surgery and refused further follow-ups.

## Discussion and conclusions

Here we report a case about a gastric band migration into distal jejunum causing small bowel obstruction more than 10 years following initial diagnosis of erosion. It highlights the importance of regular endoscopic follow-ups.

Gastric banding is a frequent intervention among those invested in bariatric surgery, albeit with variable long-term results. It is the least invasive bariatric surgical procedure but with a relatively high re-intervention rate [4]. Band-related complications are pouch dilatation, band erosions, intolerance, leakage, and slippage. A systematic review of the literature reported an incidence of erosion after LAGB of 1.46% resulting from a broad range of 0.23–32.65% [5]. Although a high frequency is mostly seen in early experience and decreases with growing routine. Early laceration of the dorsal portion of the cardia can be caused by inadvertent retrogastric tunneling, or through pulling the band with a hooked retraction device [6]. Later erosions may also be caused by chronic shear stress leading to micro-perforation or chronic overfilling provoking micro-ischemic events. Anyway, it is most likely that the etiology of most band erosion is multifactorial.

Clinically, patients with gastric erosions usually complain of newly developed upper abdominal pain and an increase in weight caused by lack of satiety. Some may also present symptoms of bowel obstruction or sepsis with peritonitis. Diagnosis is usually confirmed endoscopically, and verified through cross-sectional imaging in cases of dislocation, with a quoted time-to-event ranging between 6 and 132 months [7, 8]. The gastric band typically migrates intragastric, but several case reports have described migration into small bowel and colon [9, 10]. One case even reported a band migrating to the rectum [11].

Diversity exists regarding the optimal therapy and timing of intervention. Band removal can be achieved by surgery (open or laparoscopic) or by endoscopy. In historical reports the removal of the intragastric migrated band was obtained by laparotomy followed by a gastrotomy [12]. However, laparoscopic approaches gained more and more attention because the minimally invasive technique leaves the abdominal cavity with fewer adhesions and in consideration of the fact that a future bariatric surgery may be needed [13]. It still is the treatment of choice in emergency cases such as infection, obstruction, or intraabdominal perforation. All similar cases in the literature describing intestinal obstruction by a migrated gastric band into the jejunum describe a laparoscopic removal.

However, recent advances in endoscopic technique have allowed endoluminal division and removal of the gastric band [14]. Intragastric migration without obstruction does not need immediate intervention; therefore, an elective endoscopic treatment should be the treatment of choice.

A rather expectative approach, which we practice in our clinic, is to wait after diagnosis until the band has sufficiently perforated (at least 50%) the gastric wall in order to facilitate removal endoscopically. The band can be cut through by endoscopic laser, scissors, metallic thread, or electrosurgical devices – to mention only a few techniques – and finally retrieved by the patient’s mouth [15]. The formed capsule around the band serves temporarily as a gastric neo-wall. In this approach, regular follow-up is critical to monitor progression of band erosion in order not to miss full perforation into gastric lumen, which unfortunately happened in this case.

Removal of the band without any further bariatric surgery often results in resurgence of weight gain, deterioration of physical and psychological comorbidities, and low quality of life scores [16]. However, the immediate reinsertion of a new band after removal of the previous is associated with a high recurrence rate of erosions; whereas delayed replacement (> 3 months) as part of a two-stage procedure has been shown to yield more favorable clinical outcomes [17]. A viable alternative for patients with band erosions or intolerance may also lay in the conversion to laparoscopic Roux-en-Y gastric bypass or laparoscopic sleeve gastrectomy [18].

In our case, gastroscopic removal of the band was trialed before surgery in view of the hindered situation. Unfortunately, this was not successful and for safety reasons aborted. Conversion to gastric bypass was not performed as per an advance directive provided by the patient.

As a high-volume bariatric center, we present our first experience of this rare complication. The prolonged period between first diagnosis of band erosion and clinical manifestation of the meanwhile migrated band is remarkable. The cessation of periodical control gastroscopy was unfortunate, assuming that migration and consequent bowel obstruction could have been avoided by earlier removal of the band. Prospective and long-term follow-up is mandatory in those with partial band erosion, for whom regular endoscopic surveillance is advocated.

## Abbreviations

BMI: Body mass index
LAGB: Laparoscopic adjustable gastric band
